# Introducing PROCEED: a new service for fast registration and publication of protocols for environmental evidence syntheses, including Rapid Reviews

**DOI:** 10.1186/s13750-022-00295-7

**Published:** 2023-01-10

**Authors:** Andrew Pullin

**Affiliations:** School of Natural Sciences, Bangor University, Bangor, UK

Prospective registration of protocols for evidence syntheses is regarded as best practice in order to avoid unnecessary duplication of effort and to minimise potential for bias due to *post hoc* modifications in review conduct and bias in reporting of findings. They also provide authors and evidence users with a catalogue of review activity from which to find relevant evidence and confirm evidence gaps. Preregistration of titles and protocols is now common practice in some disciplines such as medicine and healthcare and specialist databases are provided for the global community to use. For example, PROSPERO is an international database of prospectively registered systematic reviews in health and social care, welfare, public health, education, crime, justice, and international development (https://www.crd.york.ac.uk/prospero/). In the discipline of environmental science the prospective registration and/or publication of titles and protocols is comparatively rare. This journal, founded by the Collaboration for Environmental Evidence (CEE), is an exception and since its establishment in 2011, has continued the CEE Standard of requiring the publication of protocols in the journal prior to considering publication of the subsequent Systematic Reviews and Systematic Maps. This practice has worked well in producing high quality evidence syntheses with transparent reporting of both conduct and review findings, but some authors find the process prohibitive due to time and cost constraints.

CEE, as a global network promoting and supporting the conduct of reliable evidence syntheses, now offers authors the opportunity to register protocols in its new database PROCEED (https://www.proceedevidence.info/) maintained in partnership with the Julius Kuhn Institute. PROCEED is a free service to authors and other users of the database. Authors can register protocols for Systematic Reviews, Systematic Maps, Rapid Reviews and other forms of both aggregative (e.g. meta-analyses) and configurative (e.g. bibliographic mapping) reviews. CEE has established this service with the objective of increasing rigour of evidence syntheses wherever they are published and so there is no restriction on where the resulting evidence syntheses are published. Authors can submit protocols to PROCEED using templates and guidance provided for each type of synthesis. Submissions will not be peer reviewed but will be checked by an editorial team and may be returned for revision before acceptance. All accepted protocols will be given a DoI and will be downloadable and citable.

For this journal, PROCEED provides the opportunity for authors to publish their Systematic Reviews or Maps in Environmental Evidence without the previous requirement to first publish the protocol in the journal. Authors can still submit protocols for publication in this journal if they wish but this is no longer compulsory. Instead, registration of the protocol in PROCEED is the compulsory element.

The free and rapid registration service that PROCEED offers also enables Environmental Evidence to publish Rapid Reviews. This is an important development for the journal in its objective to produce reliable and timely evidence to inform environmental policy. Rapid Reviews have become popular with decision makers where the need for evidence is urgent, and decisions need to be made in short time windows. However, CEE has recognised that methodological guidance and standards on how to conduct and report Rapid Reviews has been lacking in environmental management. We invite authors of Rapid Reviews to follow CEE’s new Guidance and Standards (https://environmentalevidence.org/information-for-authors/ - Section.10), register their protocols in PROCEED and publish their findings in Environmental Evidence.

## Data Availability

Not applicable.

